# Magnetization Transfer Imaging of Suicidal Patients with Major Depressive Disorder

**DOI:** 10.1038/srep09670

**Published:** 2015-04-08

**Authors:** Ziqi Chen, Huawei Zhang, Zhiyun Jia, Jingjie Zhong, Xiaoqi Huang, Mingying Du, Lizhou Chen, Weihong Kuang, John A. Sweeney, Qiyong Gong

**Affiliations:** 1 Huaxi MR Research Center (HMRRC), Department of Radiology, West China Hospital of Sichuan University, Chengdu, PR China; 2 Department of Nuclear Medicine, West China Hospital of Sichuan University, Chengdu, PR China; 3 Department of Neurology, West China Hospital of Sichuan University, Chengdu, PR China; 4 Department of Psychiatry, State Key Lab of Biotherapy, West China Hospital of Sichuan University, Chengdu, PR China; 5 Departments of Psychiatry and Pediatrics, University of Texas Southwestern, Dallas, Texas, USA; 6 Department of Psychology, School of Public Administration, Sichuan University

## Abstract

Magnetization transfer imaging (MTI) provides a quantitative measure of the macromolecular structural integrity of brain tissue, as represented by magnetization transfer ratio (MTR). In this study, we utilized MTI to identify biophysical alterations in MDD patients with a history of suicide attempts relative to MDD patients without such history. The participants were 36 medication-free MDD patients, with (N = 17) and without (N = 19) a history of a suicide attempt, and 28 healthy controls matched for age and gender. Whole brain voxel-based analysis was used to compare MTR across three groups and to analyze correlations with symptom severity and illness duration. We identified decreased MTR in left inferior parietal lobule and right superior parietal lobule in suicide attempters relative to both non-attempters and controls. Non-attempters also showed significantly reduced MTR in left inferior parietal lobule relative to controls, as well as an MTR reduction in left cerebellum. These abnormalities were not correlated with symptom severity or illness duration. Depressed patients with a history of suicide attempt showed bilateral abnormalities in parietal cortex compared to nonsuicidal depressed patients and healthy controls. Parietal lobe abnormalities might cause attentional dysfunction and impaired decision making to increase risk for suicidal behavior in MDD.

Suicide is a major global social and public health problem. Patients with major depressive disorder (MDD) have a 2–12% lifetime risk of committing suicide^1^,^2^. The most powerful predictor of completed suicide is a prior history of suicide attempts^3^. Thus, studying the neurobiology of depressed patients with a history of suicide attempts is a promising strategy for learning about biological factors that may confer risk for suicidal behavior, and potentially for providing an objective neurobiological marker of risk.

Structural brain abnormalities have been reported in depressed patients with suicidal behavior, including gray matter (GM) hyperintensities in the basal ganglia^4^, periventricular white matter hyperintensities (PVH)^5^ and deep white matter hyperintensities (DWMH)^6^. Abnormal GM volumes in temporal-parietal-limbic networks has been reported to be related to suicide attempts in young depressed patients^7^. Another study suggested decreased GM and white matter volume in frontal, parietal, and temporal regions, insula, lentiform nucleus, midbrain, and cerebellum compared with non-suicidal depressed patients in late-onset depression with suicidal behavior^8^.

The macroscopic findings of structural alteration in suicide attempters have been further confirmed by post-mortem studies. Schlicht et al. found that three proteins including a phosphorylated isoform of glial fibrillary acidic protein (GFAP), manganese superoxidase dismutase (SOD2) and α crystallin chain B (CRYAB) were present at greater levels in the prefrontal cortex (PFC) of suicide victims, indicating a possible link between glial integrity and suicidal behavior^9^. Decreased neuron density^10^ and protein expression of brain-derived neurotrophic factor (BDNF) in the PFC^11^,^12^, larger cell bodies of fibrous astrocytes in anterior cingulate white matter^13^, all have been described in post-mortem brain of suicide victims. However, it has been more difficult to characterize the histopathological changes *in vivo* that underlie the macroscopic abnormalities. Postmortem findings point to alterations that could be identified with MRI studies, but that which might be difficult to identify with standard morphometric imaging. Techniques are needed that have increased neuropathological sensitivity to relatively subtle macromolecular changes.

A technique that is particularly interesting in this context is magnetic transfer imaging (MTI). MTI creates a contrast between tissues by exploiting the phenomenon of magnetization exchange between the spins of free water and water bound to macromolecules. The efficiency of these exchange phenomena is measured by the magnetization transfer ratio (MTR), which depends on both the amount and states of macromolecules^14^. MTI is highly sensitive to white matter abnormalities, e.g., in schizophrenia^15^, depression^16^,^17^ and multiple sclerosis (MS) even when conventional MRI is negative^18^. Lower MTR in gray matter is believed to be associated with abnormalities of cell membrane proteins and phospholipids^19^. Furthermore, Wallerian degeneration triggered by distant axonal damage and microscopic lesions have also been implicated as a mechanism underlying cortical MTR reductions^19^.

The suitability of MTI for the investigation of subtle biophysical alterations with macromolecular concentration changes in treatment-resistant depression has been illustrated by our group^16^. The purpose of this study was to use MTI to explore and characterize further the neuropathological abnormalities *in vivo i*n MDD patients with history of a suicide attempt relative to other depressed patients and healthy controls.

## Results

### Demographic and Clinical Comparisons

Table 1 presents demographic and clinical characteristics of study participants. Gender, age, education and race/ethnicity did not differ significantly among the three subject groups. HAM-D scores of depressed patients did not differ significantly between suicide attempters and non-attempters, but illness duration was longer in depressed non-attempters compared to suicide attempters (p < 0.05).

### Voxel-Based Analysis

**Figure 1:**
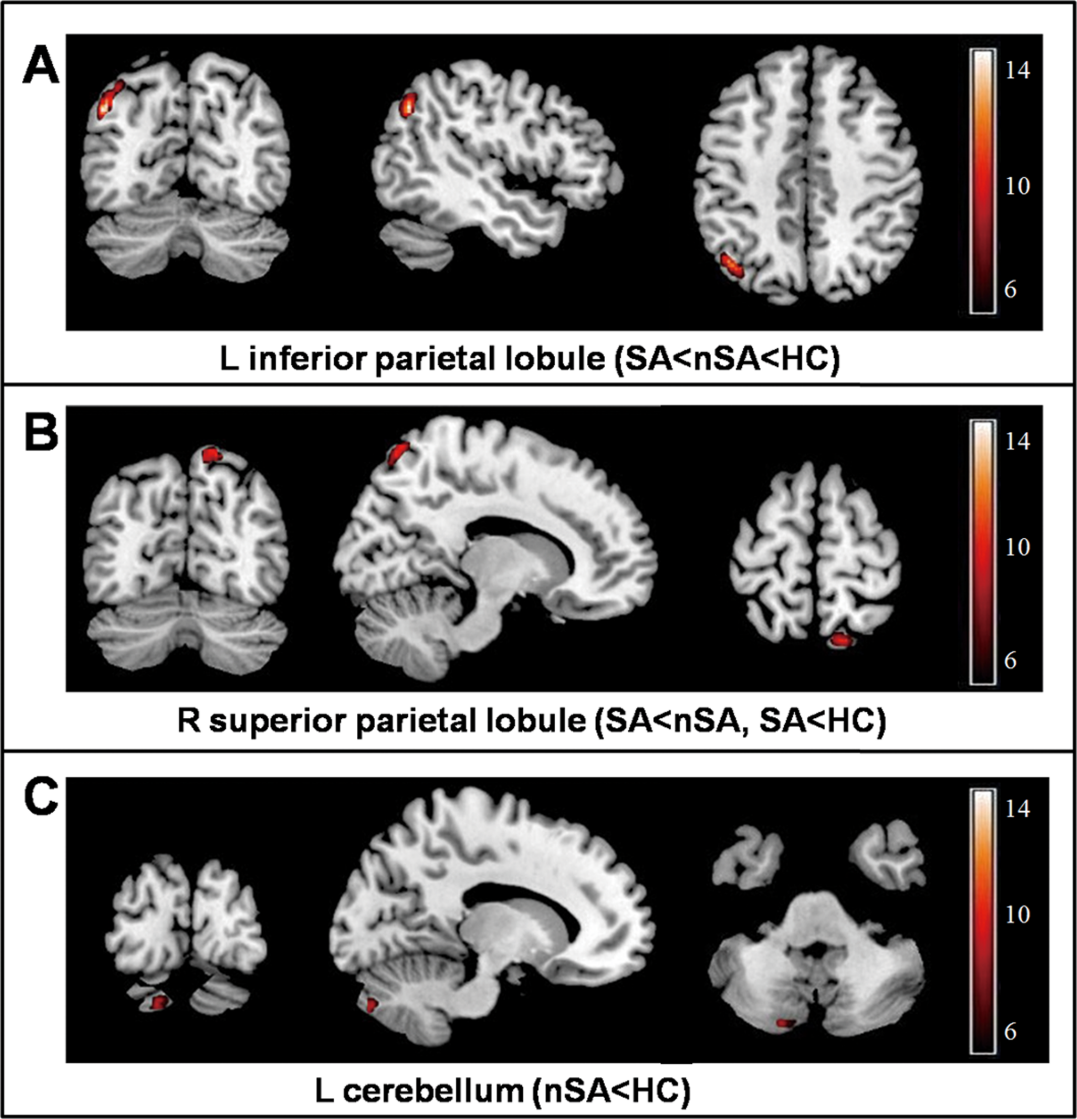
Magnetization transfer ratio differences in voxel-based analysis comparisons among major depressive disorder patients, with and without a history of suicide attempts, and healthy comparison subjects^a^. ^a^ Images are presented in neurological convention. Suicide attempters showed reduced MTR in (A) the left inferior parietal lobule and (B) the right superior parietal lobule relative to non-attempters and healthy controls. Non-attempters showed reduced MTR in (A) the left inferior parietal lobule and (C) the left cerebellum (posterior lobe) relative to healthy controls. Statistical inferences were made with a voxel-level statistical threshold of p < 0.05 (corrected). Abbreviations: HC = healthy comparison subjects; L = left; MTR = magnetization transfer ratio; nSA = depressed patients without a history of suicide attempts; R = right; SA = depressed patients with a history of suicide attempts.

**Figure 2:**
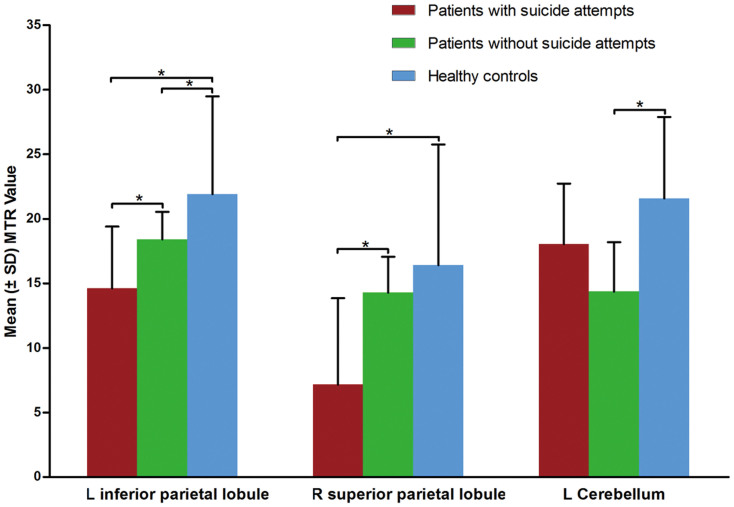
Magnetization transfer ratio in major depressive disorder patients, with and without a history of suicide attempts, compared to healthy subjects^a^. ^a^ Brain regions differed significantly among the three groups in the left inferior parietal lobule and right superior parietal lobule between suicide attempters and healthy controls and in the left inferior parietal lobule and left cerebellum (posterior lobe) between non-attempters and healthy controls. Abbreviations: L = left; MTR = magnetization transfer ratio; R = right. * p < 0.05.

The three groups had significant differences of MTR in the left inferior parietal lobule, right superior parietal lobule and left cerebellum (posterior lobe) (Figure 1, Table 2). Post-hoc t tests showed that suicide attempters had reduced MTR in the left inferior parietal lobule and right superior parietal lobule relative to both non-attempters (p = 0.005 and p < 0.001, respectively) and healthy controls (p < 0.001 and p = 0.003, respectively). Non-attempters also showed reduced MTR in the left inferior parietal lobule (p = 0.012), as well as a reduction in left cerebellum (posterior lobe) (p < 0.001) relative to healthy controls. No other significant group differences were identified. Figure 2 illustrates the changes in the MTR values in these affected regions. No association was found between regional MTR values and HAM-D scores or illness duration.

### Region-of-Interest Analysis

Region-of-interest analysis revealed no significant group differences of MTR in the head of the caudate nucleus (F = 0.611, df = 2, p = 0.546 left caudate; F = 0.34, df = 2, p = 0.713 right caudate, see Supplementary Figure S1 online).

## Discussion

To our knowledge, this is the first MTI study to compare untreated adult patients with major depressive disorder with and without a history of suicide attempts using a voxel-based analysis. The main findings are that suicide attempters showed reduced MTR in the left inferior parietal lobule and right superior parietal lobule relative to non-attempters and healthy controls. Non-attempters showed reduced MTR in the left inferior parietal lobule and left cerebellum (posterior lobe) relative to healthy controls.

The MT signal is largely dependent on the macromolecular density of cell membranes and phospholipids, and grey matter MTR reductions in the parietal lobe in the patients we observed likely reflect decreases in the size and number of neurons and dendritic density. One possible mechanism for these effects is that stress-induced hyperactivity of the hypothalamic–pituitary–adrenal axis may lead to cell damage through glucocorticoid-mediated glutamatergic neurotoxicity and decreased astrocytic activation, with reduced uptake of glucose and altered neuronal metabolism resulting in reduced neuronal volume and dendritic arborization^20^. Consistent with this possibility, lower regional cerebral blood flow (rCBF) in the parietal lobe has been reported in depressed suicide attempters compared to healthy controls^21^.

The suicide attempters had a significantly lower MTR than non-attempters and healthy controls in the left inferior parietal lobule and right superior parietal cortex, while non-attempters also showed a lower MTR than healthy controls in the inferior parietal lobule, suggests that a biophysical abnormality in parietal cortex in depression is amplified in patients with a history of suicidal behavior. The lower MTR in left inferior parietal lobule in SA group may be due to the combined effects of depression and suicidal behavior. The inferior parietal lobule is a hub region of the ventral attention network that interrupts and resets attention to behaviorally salient stimuli. Moreover, this brain region is involved in action organization^22^, decision making and predictions of reward when outcomes of behavioral choices are uncertain^23^. All of these processes are impaired in depression and have been related to suicidal behavior^24^,^25^. Generally, people make decisions based on prediction of rewards at different time scales. In many cases, the choice for a greater longer-term positive outcome is more appropriate than a choice for lesser immediate reward^26^. Suicide attempters have been shown to have a reduced ability to forecast positive outcomes of their actions, resulting in disadvantageous choices and a bias toward making choices likely to yield immediate rather than greater longer term reward^25^.

Previous studies have noted structural and functional abnormalities of the left inferior parietal lobule in relation to MDD and a history of suicide attempts. Increased resting-state regional homogeneity (ReHo)^27^, reduced MTR^28^ and white matter volumes^29^ in the inferior parietal lobule have been reported in MDD patients compared to healthy controls. Moreover, decreased volume of gray matter in the inferior parietal lobule has been reported in late-onset geriatric depression with a history of suicide attempts compared with other depressed patients^4^. One SPECT study showed that depressed suicide attempters had a lower regional cerebral blood flow (rCBF) in the inferior parietal lobule compared to healthy controls when concentrating^21^. In bipolar disorder and schizophrenia patients with a history of suicidal behavior, structural abnormalities of left parietal lobe have also been reported^30^. The question of whether the parietal abnormalities might be related to suicidal behavior across major psychiatric disorders beyond MDD, and whether these could also be detected with MTI, needs to be further studied.

We also identified a lower MTR in the right superior parietal lobule of suicide attempters compared to non-attempters and healthy controls. Similar to the inferior parietal lobule, the superior parietal lobule is a core region of the dorsal attention network, which maintains the locus of attention in the face of distraction, selects stimuli according to prior information or goals, and regulates responses^31^. Event-related fMRI studies have shown enhanced activation in the parietal lobe in attention switching tasks^32^. Deficits in attentional control are common in psychological disorders, such as posttraumatic stress disorder^33^ and depression^34^. Impaired attention control has also been noted in suicide attempters, especially if provocative distractors (i.e., suicide-related words) are used^35^. Parietal dysfunction in suicide attempters may lead to an impaired ability to redirect attention, which may predispose suicide attempters to persistent rumination and associated emotional states^36^,^37^, causing them to attempt to take their lives based on a narrow view of their current life difficulties, their coping options and future possibilities. Therefore, the biophysical alterations that decrease MTR in the parietal lobes of depressed patients with suicide attempts suggest that attentional network and decision making abnormalities may interact in shaping the risk of committing suicide in MDD. Further work is needed to investigate the relationship among attentional dysfunction, parietal lobe anatomy and physiology, and impaired decision making in suicidal behavior.

We found that relative to healthy controls, non-attempters had lower MTR in the left cerebellum (posterior lobe). There was no significant difference between suicide attempters and comparison subjects in this region. A study using repetitive transcranial magnetic stimulation reported that the inhibition of cerebellar function resulted in mood dysregulation and increased negative moods in healthy subjects^38^. This is consistent with a body of literature showing a role of cerebellum in emotion modulation^39^. The abnormality in MTR of the left cerebellum is consistent with a previous report of reduced GM volume in the cerebellum in MDD patients^40^.

ROI analysis revealed no significant group differences in the head of the caudate nucleus, failing to replicate a prior report that a biophysical abnormality in the caudate nucleus is related to suicidal behavior or depression^41^. This is consistent with a previous study that showed unchanged density of caudate nucleus dopamine uptake sites in depressed suicide completers^42^. However, a smaller right caudate nucleus has been reported in suicide attempters^43^ as has decreased gray matter density in the caudate in MDD patients at high risk for suicide compared to non-high risk patients^44^. These results differ presumably as a result of inter-study differences in clinical characteristics of the participants, time between attempt and scanning, and technical characteristics of image acquisition and analysis. Thus while our findings do not show caudate pathology, further work is needed to investigate the role of striatal changes in suicidal behavior.

Studies of depressed patients with a history of suicide attempts using different imaging modalities have revealed significant findings. Two independent DTI studies and an fMRI study identified the anterior limbs of the internal capsule^45^, dorsomedial prefrontal cortex^46^ and superior temporal gyrus^47^ as anatomical sites with pathology related to suicidal behavior. A voxel-based morphometry (VBM) study demonstrated that suicidal behavior in late-onset MDD was associated with decreased gray and white matter volume in frontal, parietal, and temporal regions, as well as in insula, lentiform nucleus, midbrain, and cerebellum^8^. As different imaging methods are more sensitive to others in detecting different specific alterations, the emerging results documenting different alterations in different regions does not necessarily indicate a discrepancy in findings. Rather it may reflect the biological complexity of the problem with different brain regions showing alteration depending on the imaging method used and patient population characteristics. Here, we observed MTR reductions in the parietal lobe and cerebellum in medication-free MDD patients with a history of suicide attempts. Future multimodal imaging studies may provide useful insights to facilitate understanding of different pathological processes in different regions that may increase risk for suicidal behavior.

Our study has certain limitations. First, the group of suicide attempters was relatively small which may have limited statistical power to identify less robust effects. Second, while patients were medication free for at least two weeks before the MRI study, prior treatment might have impacted our findings. Third, the study did not have the power to examine variability in brain anatomy with regard to multiple potentially relevant dimensions such as the particular method of suicide attempts, number of previous attempts and their medical lethality, age, sex, etc. that might moderate relations between suicide risk and alterations in brain structure. Fourth, the question of whether lower MTR in the parietal lobe is specific to suicidality in MDD can not be answered by the present study. However, as alterations in this region have been reported in other disorders in relation to suicidal behavior^30^, effects in this region might not be diagnostically specific. Fifth, the specific neurobiological causes of the identified MTR alterations remain to be determined. Finally, the present results cannot definitively establish the direction of causality (i.e., whether a reduced MTR causes a predisposition to suicide attempts, or vice versa). This question requires longitudinal studies to follow depressed patients prospectively in order to be able to compare patients before and after a suicide attempt.

In conclusion, our MTI findings indicate that there are biophysical abnormalities related to suicidal behavior in parietal cortex in depressed patients. Attentional dysfunction and impaired decision that can result from parietal abnormalities might contribute to suicidal behavior in the context of MDD. Studies working to directly link parietal lobe alterations with such neurobehavioral deficits in suicidal patients are needed to support this hypothesis, and link regional neocortical changes to specific psychological patterns known to be related to suicidal behavior. Future studies with longitudinal designs, large samples and comparison groups of patients with other psychiatric disorders associated with increased suicide risk are needed to better characterize the neurobiological mechanisms of suicidal behavior and their specificity to MDD.

## Methods

### Subjects

We recruited patients with MDD from the Department of Psychiatry at West China Hospital of Sichuan University. All patients were assessed by an experienced psychiatrist and met DSM-IV criteria for MDD^48^. Patients were medication-free for at least 2 weeks before MRI scanning and had a 17-item Hamilton Rating Scale for Depression (HAM-D)^49^ total score of 18 or higher on the day of their scan. Patients were divided into two groups: suicide attempters with a history of at least one suicide attempt, defined as a self-destructive act causing physical harm with some degree of intent to die^36^ within one month prior to magnetic resonance scanning, and non-attempters without such a history. Patients were not considered to have a history of suicide attempts if they had only suicidal thoughts, a suicide plan or self-injurious behaviors without suicidal intention or ideation. Exclusion criteria were any psychotic disorders other than MDD; significant medical or neurological illness, including any history of significant head trauma with loss of consciousness; substance or alcohol abuse or dependence within the past 12 months, and any likelihood that the suicide attempt itself would have caused structural brain abnormalities. Healthy comparison subjects were recruited from the local area by poster advertisement and assessed with the Structured Clinical Interview for the DSM-IV. Exclusion criteria included the same medical and psychiatric factors used to recruit patients, as well as any DSM-IV Axis I disorder, or known history of significant psychiatric illness or suicide among first-degree relatives. All participants were of Chinese Han nationality.

Independent-sample t test and ANOVA (followed by Tukey post-hoc pairwise comparisons if significant) were used to compare quantitative variables across groups. Qualitative variables were compared using a chi-squared test. The threshold for these statistical analyses was set at p < 0.05. Statistical analyses were carried out using SPSS.19 (SPSS, Inc., Chicago).

The West China Hospital Clinical Trials and Biomedical Ethics Committee of Sichuan University approved our study protocol, and written informed consent was obtained from all participants. The methods were carried out in accordance with the approved guidelines.

### MRI Acquisition

Images were acquired using a 3.0 Tesla General Electric magnetic resonance scanner (EXCITE, General Electric Medical Systems, Milwaukee, Wisconsin, USA). Participants were fitted with soft ear plugs, positioned comfortably in the coil and instructed to relax and keep still. We minimized head motion using foam pads. Whole brain MT images were acquired using a 3-dimensional fast low angle shot sequence. One acquisition was performed with, and another without, the magnetization saturation pulse at 1.5 kHz off-resonance. In this way, MT-weighted and non-MT-weighted images were generated separately. The other sequence parameters were as follows: TR/TE = 37/5 ms; flip angle (FA) = 15°; 50 contiguous axial slices with slice thickness = 3 mm; Field of View (FOV) = 24 × 24 cm^2^; and data matrix = 320 × 192. High-resolution three-dimensional T1-weighted images were acquired using a spoiled gradient recalled sequence (repetition time = 8.5 ms, echo time = 3.4 ms, fractional anisotropy = 12°, 156 axial slices with thickness of 1 mm, axial field of view = 240 × 240 mm, data matrix = 256 × 256).

### Image Processing

MR images from all the subjects were first reviewed by a neuroradiologist to ensure that there were no structural abnormalities or data quality flaws. Data processing and analysis were carried out using the statistical parametric mapping software SPM8 (Wellcome Trust Centre for Neuroimaging). For each subject, the MT-weighted and non-MT-weighted images were first co-registered using a mutual information registration algorithm. MTR was then calculated on a voxel-by-voxel basis as follows: MTR = (M_0_-M_s_)/M_0_ × 100, where M_0_ and M_s_ are the signal intensities without and with the saturation pulse applied. Because the non-MT images are partially T1-weighted, we directly normalized them to the MNI T1W template and then used the transformation parameters to normalize the co-registered MTR map. The normalized non-MT images were skull-stripped using the brain extraction tool (BET, http://www.fmrib.ox.ac.uk/fsl/bet/) and were then used as masks to remove non-brain tissues on the normalized MTR maps. Finally, MTR maps were smoothed with a Gaussian kernel of 6-mm full-width half-maximum.

### Voxel-Based Analysis

MTR maps were compared among the three groups using analysis of covariance (ANCOVA) with age and sex as covariates. Correction for multiple comparisons was determined by Monte Carlo simulations (with the following parameters: individual voxel p value = 0.005, 1,000 simulations, FWHM = 6 mm), applied with the Resting-State fMRI Data Analysis Toolkit (REST) of the AlphaSim program^50^, to determine a corrected significance level of p < 0.05 for a minimum cluster size of 86 voxels. For clusters identified in the ANCOVA, we performed follow-up between-group voxel-wise t tests in regions with overall group differences to identify pair-wise group differences using the same thresholding method. Montreal Neurological Institute coordinates were transformed to Talairach coordinates using MNI2tal (http://imaging.mrc-cbu.cam.ac.uk/downloads/MNI2tal/). To quantify changes in the affected regions, MTR values were extracted using a volume-of-interest approach in SPM. We conducted correlation analyses between the average regional values in these regions with HAM-D score and illness duration.

### Region-of-Interest Analysis

**Figure 3:**
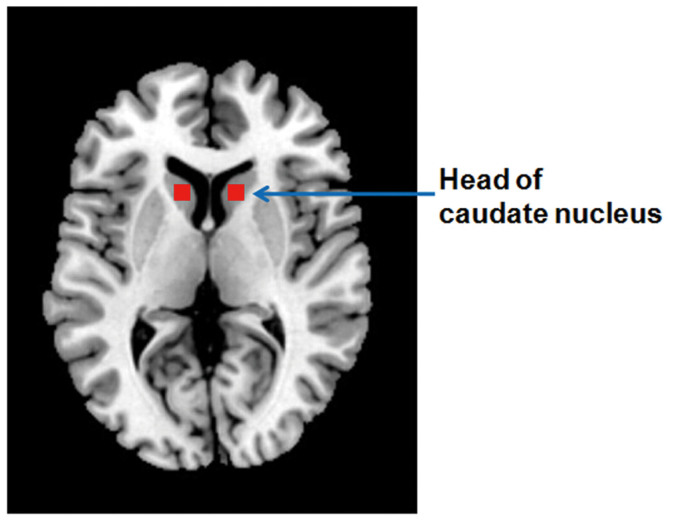
Regions of interest (head of bilateral caudate nucleus) for magnetization transfer ratio analysis.

In a secondary analysis, ITK-SNAP (www.itksnap.org)^51^ was used to obtain bilateral measures of the head of the caudate nucleus. This was done based on a previous MT study reporting focal biophysical abnormalities in this region in depression^41^. The slice displaying the most anterior margin of genu of the corpus callosum (Montreal Neurological Institute (MNI) coordinates: [1, 32, 6]) was chosen as the reference slice for placing the ROI, since this landmark could be easily and consistently identified across subjects (see Figure 3). We used constant volumes 72 mm^3^ for the head of caudate nucleus in the MTR maps of all subjects and the MTR values were extracted bilaterally and compared across groups using univariate analysis of covariance controlling for age and sex.

## Author Contributions

Z.Y.J. and Q.Y.G. conceptualized the project. Z.Q.C. and H.W.Z. designed the protocol. Z.Q.C., Z.Y.J. and J.A.S. wrote the main manuscript text. Z.Q.C., H.W.Z., J.J.Z., W.H.K. and X.Q.H. performed the experiments. Z.Q.C., M.Y.D. and L.Z.C. conducted the statistical analyses. All authors reviewed the manuscript. Z.Q.C., Z.Y.J. and Q.Y.G. revised the manuscript.

## Supplementary Material

Supplementary Information Supplementary Information

## Figures and Tables

**Table 1:** Demographic and clinical characteristics of patients with major depressive disorder, with or without a history of suicide attempts, and of healthy controls^a^

Characteristic	Major Depressive Disorder Patients		Healthy	*P*
	Non-attempters (N = 19)	Suicide Attempters (N = 17)	Controls (N = 28)	value
Age (years), mean (SD)	38 (13.2)	36 (12.8)	32 (10.4)	0.249
Female, N (%)	7 (36.8)	12 (70.6)	17 (60.7)	0.103
Education (years), mean (SD)	12.8 (3)	11.2 (4.5)	12.1 (4)	0.49
Illness duration (months), mean (SD)	111 (99)	38 (55)		0.009
HAM-D score, mean (SD)	22.6 (4.3)	25 (4.2)		0.096
Race/ethnicity, N (%)				
Asian/Chinese	19 (100)	17 (100)	28 (100)	>0.999

^a^Statistical significance between both patient groups was observed for disease duration alone (p < 0.05). No significant differences were found between either group of patients and healthy controls.

Abbreviations: HAM-D = Hamilton Rating Scale for Depression; SD = standard deviation.

**Table 2:** Differences in the magnetization transfer ratios among depressed patients, with and without a history of suicide attempts, and healthy subjects

				Comparison^c^		
	Talairach	Cluster Size				
Anatomical Region	Coordinate (x, y, z)^a^	(Voxels)	F^b^	nSA < HC	SA < HC	SA < nSA
L inferior parietal lobule	−48,−64,44	333	13.99	3.99	5.13	3.99
R superior parietal lobule	16,−67,66	131	11.85		4.4	4.16
L cerebellar posterior lobe	−2,−87,−28	110	9.33	4.17		

^a^Talairach location is the centroid of the cluster.

^b^All effects survived a voxel-wise statistical threshold (p < 0.05), as corrected for multiple comparisons using AlphaSim program.

^c^Data indicate voxel-wise t values; the magnetization transfer ratio is lower in the former than in the latter.

Abbreviations: HC = healthy controls; L = left; nSA = depressed patients without a history of suicide attempts; R = right; SA = depressed patients with a history of suicide attempts.
