# Supplementary material for: Magnetization Transfer Imaging of Suicidal Patients with Major Depressive Disorder
Source: Sci Rep. 2015 Apr 8;5:9670. doi: 10.1038/srep09670 (PMC4389668; doi:10.1038/srep09670)
Supplement: Supplementary Information [file srep09670-s1.pdf]

Magnetization Transfer Imaging of Suicidal Patients with Major Depressive Disorder

Ziqi Chen[a], Huawei Zhang[a], Zhiyun Jia[a,b]※, Jingjie Zhong[c], Xiaoqi Huang[a], Mingying Du[a], Lizhou Chen[a], Weihong Kuang[d], John A. Sweeney[e], Qiyong Gong[a,f]※

Supplementary Figure S1. Scatter plots of magnetization transfer ratio by region-of-interest (head of caudate nucleus) among major depressive disorder patients with and without a history of suicide attempts and healthy controls[a]

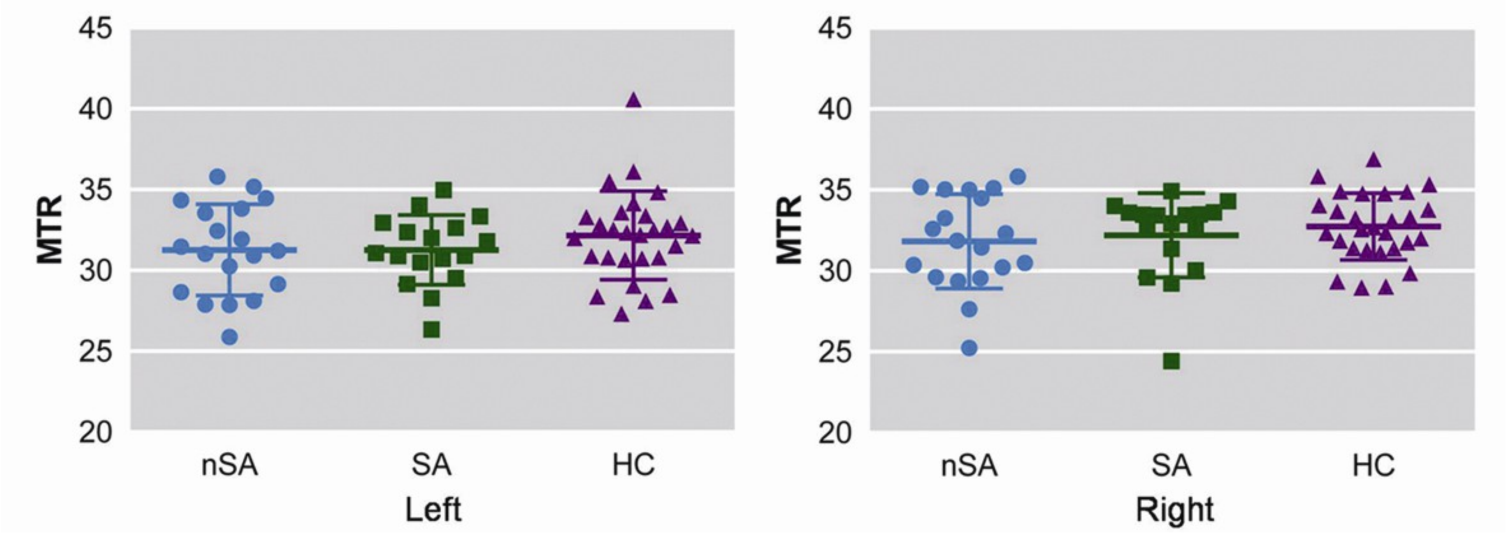

[a] No significant difference was observed in the head of left ($p = 0.546$) or right ($p = 0.713$) caudate nucleus among patients with major depressive disorder with (SA) and without (nSA) a history of suicide attempts and healthy controls (HC), revealed by region-of-interest analysis. The bold horizontal line represents the mean; error bars represent one standard deviation.
